# A Cellular Automaton-Based Computational Model for Fluid Shear Stress-Induced Differentiation and Migration of Osteoprogenitor Cells in a Microfluidic Chip

**DOI:** 10.3390/bioengineering13070813

**Published:** 2026-07-16

**Authors:** Di Jiang, Yujiang Li, Xinyao Qian, Lingbo Lu, Mao Liu, Lizhe Xie, Bin Wu, Bin Yan

**Affiliations:** 1 College of Mechanical and Electronic Engineering, Nanjing Forestry University, Nanjing 210037, China; 2 Department of Orthodontics, School of Stomatology, Nanjing Medical University, Nanjing 210029, China; 3 State Key Laboratory Cultivation Base of Research, Prevention and Treatment for Oral Diseases, Nanjing Medical University, Nanjing 210029, China; 4 Jiangsu Province Engineering Research Center of Stomatological Translational Medicine, Nanjing 210029, China; 5 Jiangsu Key Laboratory for Design and Manufacture of Micro-Nano Biomedical Instruments, School of Mechanical Engineering, Southeast University, Nanjing 211189, China

**Keywords:** fluid shear stress, osteogenic differentiation, microfluidic chip, cellular Potts model

## Abstract

This study evaluates the mechanobiological responses of MC3T3-E1 cells to fluid shear stress utilizing a coupled CFD-CPM mesoscale framework. Computational fluid dynamics was utilized to calculate the distribution of fluid shear stress within the culture chamber, which was subsequently mapped onto a discrete system of lattices. The cellular Potts model was employed to simulate behaviors of the cells governed by rules for proliferation, migration, contact inhibition, and osteogenic differentiation. To accurately reflect developmental stages, the computational workflow dictated that the cells complete the phase of growth prior to the initiation of differentiation. Evaluations demonstrated that the culture region formed a relatively uniform plateau of shear stress. Within an optimal range, fluid shear stress accelerates the transition of these cells into mature osteoblasts. Furthermore, staining for alkaline phosphatase revealed responses of osteogenic differentiation strictly correlated with the local distribution of fluid shear stress. Ultimately, this study establishes a visualized framework of mesoscale modeling to analyze the collective behavior of osteoblasts under mechanical stimulation in microfluidic environments, demonstrating the feasibility of predicting subsequent extracellular matrix mineralization and providing valuable insights into the dynamic evolution of bone remodeling.

## 1. Introduction

Osteoblasts (OBs) maintain bone homeostasis by secreting proteins to synthesize the matrix during remodeling [1,2,3]. This process is highly mechanosensitive. Mechanical loading deforms trabecular cavities, generating pressure gradients and interstitial fluid flow that produce fluid shear stress (FSS) [4]. Physiological FSS activates osteogenic signaling pathways and promotes OB-mediated bone formation. Understanding this mechanism provides critical insights into osteoporosis and bone remodeling [5,6,7].

Traditionally, macroscopic continuum models have been widely applied to describe collective dynamics in active biological systems [8,9,10,11]. However, they struggle to capture individual heterogeneity and discrete interactions, particularly under low-density conditions. Additionally, solving their nonlinear equations is computationally expensive [12]. Relying solely on in vitro experiments is also challenging due to complex interacting variables and condition-dependent accuracy [13].

Consequently, discrete models like cellular automata (CA) offer computationally efficient alternatives for simulating multicellular tissue dynamics [14]. CA models have been effectively utilized to simulate diverse biological processes, including cellular differentiation [15], thymocyte migration [16], skeletal muscle stem cell regeneration [17], microfluidic organ-on-chip simulations [18], and fibroblast density variations in diseases [19]. Collectively, these studies demonstrate the efficacy of CA as a robust tool for investigating in vitro cell population dynamics.

However, conventional CA models struggle with coupled continuous physical fields. The cellular Potts model (CPM) addresses this by integrating energy constraints into the CA lattice [20]. CPM naturally incorporates local pressure and maps external continuous fields onto discrete lattices. This enables direct simulations of mechanobiological behaviors like embryogenesis and tumor growth [21,22].

In parallel, microfluidic organ-on-chip systems have become indispensable for reconstructing in vitro organ models. In bone research, microfluidic chips have successfully simulated bone cellular microenvironments [23] and quantified OB calcium dynamics under FSS [24]. These devices streamline mechanical stimulation experiments, providing valuable platforms for investigating OB differentiation and migration.

Although individual-based discrete models have been widely used in cell dynamics, existing computational models rarely integrate continuous macroscopic computational fluid dynamics (CFD) fields with the microscopic CPM.

In this study, a CPM coupled with FSS effects was developed to investigate the influence of this mechanical stimulation on osteoprogenitor cells. Model parameters were derived from literature and CFD analyses, and the framework was experimentally validated using a microfluidic platform. This computational approach provides a mesoscale mechanistic explanation for FSS-regulated cell proliferation, differentiation, migration, and bone mineralization, overcoming the limitations of conventional experimentally intensive methods.

## 2. Materials and Methods

### 2.1. Design and Fabrication of Microfluidic Chips

**Figure 1:**
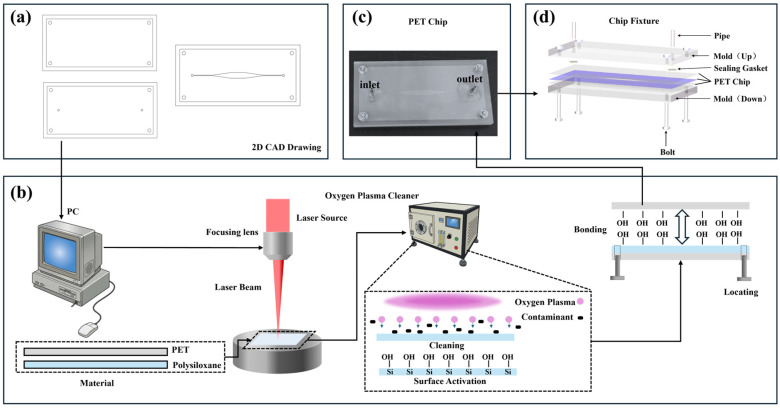
(**a**) Microfluidic chip design. (**b**) Chip fabrication process including laser machining and bonding. (**c**) Image of fabricated and sterilized microfluidic chip. (**d**) Exploded view of chip and fixture.

The microfluidic chip was fabricated using a thin-film manufacturing process (Figure 1a). The upper and lower polyethylene terephthalate (PET) films were designed with functional holes for fluidic access and alignment. The intermediate polysiloxane layer (Dudao United Chemical Company, Shenzhen, China) was patterned to form the core microchannel structure, comprising a culture chamber 4 mm in width and 50 μm in height.

Following the fabrication procedure depicted in Figure 1b, the engineering design files were imported into the processing software of an ultraviolet laser cutting machine (TH-UV200A, Suzhou Tianhong; 10 W, 355 nm laser source, Suzhou, China). The designated regions of the material were ablated to define the chip pattern. The processed layers were subsequently treated in an oxygen plasma cleaner (PDC-002, Harrick Plasma, Ithaca, NY, USA) for 3 min to remove surface contaminants and organic residues, while simultaneously introducing functional groups onto the material surfaces. Alignment pins were inserted through the circular holes located at the four corners of the three layers to ensure precise alignment. The layers were then bonded together to form a robust interface, thereby ensuring the sealing integrity of the internal microchannels. The fabricated chip is shown in Figure 1c. Finally, the chip was mounted into a fixture and secured with bolts to complete the device assembly (Figure 1d).

### 2.2. CFD Analysis of Flow Field in Microchannels

**Figure 2:**
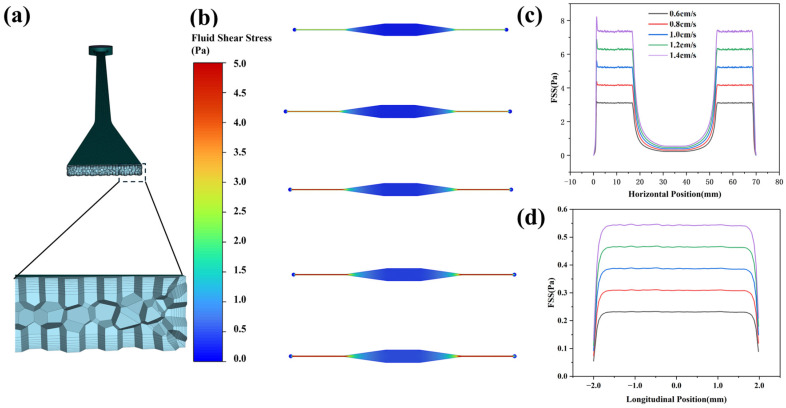
(**a**) Mesh and boundary layer. (**b**) Nephogram of bottom shear stress in the chip at 0.6–1.4 cm/s. (**c**) Shear stress distribution along the bottom centerline of the chip along the flow direction. (**d**) Fluid shear stress (FSS) distribution along the chip bottom centerline perpendicular to the flow direction.

To investigate the distribution of FSS and ensure an appropriate mechanical environment for the cells, a CFD analysis was conducted. The fluid was modeled as an incompressible, steady-state flow, with a density of 1.009 g/cm^3^ and a dynamic viscosity of 0.93 mPa·s. Boundary conditions included an inlet velocity of 0.6 cm/s, an outlet pressure of 101.325 kPa (standard atmospheric pressure), and a no-slip condition at the walls. The maximum calculated Reynolds number within the channel was 4.05, confirming the presence of a typical laminar flow regime. The computational domain was discretized using a triangular mesh with element sizes ranging from 0.2 to 6 μm. Furthermore, an eight-layer boundary layer mesh was generated near the inner walls to capture the steep velocity gradients (Figure 2a). The steady-state flow field was obtained by solving the Navier–Stokes equations using a fully coupled approach (Figure 2b). The results demonstrated that although relatively high shear stress (>2 Pa) occurred within the narrow inlet and outlet channels, an exceptionally broad and uniform shear stress plateau (0.5–1.2 Pa) formed in the central region of the culture chamber along the flow direction (Figure 2c). Moreover, the transverse distribution perpendicular to the flow direction exhibited a highly uniform profile (Figure 2d). This uniform distribution falls within the optimal range for stimulating osteogenic differentiation [5,25,26].

### 2.3. Microfluidic Cell Culture and Osteogenic Evaluation

The osteoprogenitor cells (MC3T3-E1) used in this study were provided by the Institute of Stomatology at Nanjing Medical University. Only cells between passages 5 and 10 were used. The cells were routinely cultured in 60 mm dishes containing a complete growth medium composed of α-MEM supplemented with 10% FBS and 1% penicillin-streptomycin solution. The culture medium was replaced every three days. The fluid perfusion system, consisting of a commercial syringe pump, disposable sterile syringes, silicone tubing, and a buffer reservoir, was sterilized prior to cell seeding. Specifically, the microfluidic chip was sterilized via injection of a 75% ethanol solution, followed by exposure to ultraviolet radiation for 30 min and a 12 h undisturbed incubation period. Prior to use, the assembled chip was repeatedly rinsed with phosphate-buffered saline (PBS) and coated with a 0.1 mg/mL poly-L-lysine (PLL) solution for 1 h to enhance cell adhesion.

Upon reaching approximately 80% confluence, the MC3T3-E1 cells were detached using a 0.25% trypsin-EDTA solution and diluted to a concentration of 1 × 10^6^ cells/mL. The cell suspension was injected into the microfluidic chip and incubated overnight at 37 °C in a humidified atmosphere containing 5% CO_2_ to ensure sufficient cell adhesion. After a three-day culture period to allow for cell growth, the culture medium was replaced with an osteogenic induction medium. This medium was supplemented with 50 μg/mL ascorbic acid, 10 mM β-glycerophosphate sodium, and 10 nM dexamethasone to induce osteogenic differentiation. Intermittent dynamic perfusion was applied at a flow rate of 7.2 μL/min for 1 h per day over a 7-day period. A conventional static culture control group without FSS stimulation was concurrently established in 60 mm dishes for 7 days.

Following the induction period, the differentiated cells were evaluated using an ALP staining kit. The chip was rinsed with PBS, fixed with a 4% paraformaldehyde solution for 30 min, and subsequently rinsed with Tris-buffered saline. The staining solution was added, and the chip was incubated at 37 °C for 2 h until clear color development was observed.

### 2.4. Model Design

#### 2.4.1. FSS-Coupled CPM Framework

To simulate the evolution of cells under FSS, a cross-scale coupled CPM strategy was adopted. The physical space of the system is discretized into a regular lattice. Each lattice site is assigned a specific state: medium (0), wall (1), osteoprogenitor cell (2), osteoblast (3), or mineralized matrix (4). Apoptosis or cell detachment induced by excessive FSS is modeled as a state transition to the culture medium (0). The morphological evolution of the cells is driven by the minimization of an effective Hamiltonian energy (*H*), which represents constraints of perimeter (*H_p_*), constraints of area (*H_s_*), contact energy (*H_c_*), and specific external energy (*H_e_*) [20]:

(1) $$H=H_{p}+H_{s}+H_{c}+H_{e}$$

where *H_p_*, *H_s_*, *H_c_* and *H_e_* denote the perimeter constraint, area constraint, interfacial contact energy, and specific external energy, respectively. Their specific forms are:

(2) $$H_{p}=\sum_{\sigma }\lambda_{p}{\left(p(\sigma )-P_{\left(\sigma \right)}\right)}^{2}$$

(3) $$H_{s}=\sum_{\sigma }\lambda_{s}{\left(s(\sigma )-S_{\left(\sigma \right)}\right)}^{2}$$

(4) $$H_{c}=\sum_{\langle i,j\rangle }J(\sigma_{i},\sigma_{j})(1-\delta_{\sigma_{i},\sigma_{j}})$$

where *λ_p_* and *λ_s_* are stiffness coefficients; *p*(*σ*) and *s*(*σ*) are the current perimeter and cell area, respectively; *P*_(*σ*)_ and *S*_(*σ*)_ are their target values; *J*(*σ_i_,σ_j_*) is the contact energy coefficient matrix between neighboring lattice sites; and *δ_σi,σj_* represents the Kronecker delta function, which ensures that the interfacial contact energy is calculated exclusively at cell boundaries.

The external energy term incorporates the fluid-induced force, enabling the cells to respond to local shear stress. Subsequent state updates of the lattice are governed by the Metropolis criterion, where the transition probability *P* is determined based on the total energy change (Δ*H*) in the system:

(5) $$P(\Delta H)=\left\{\begin{array}{ll} 1 & \mathrm{if}\;\Delta H<h\\ e^{-(\Delta H-h)/T} & \mathrm{otherwise} \end{array}\right.$$

where *h* denotes the yield energy threshold for state transitions, and *T* represents the activity of membrane edge fluctuations, reflecting the intrinsic motility of the cells.

**Figure 3:**
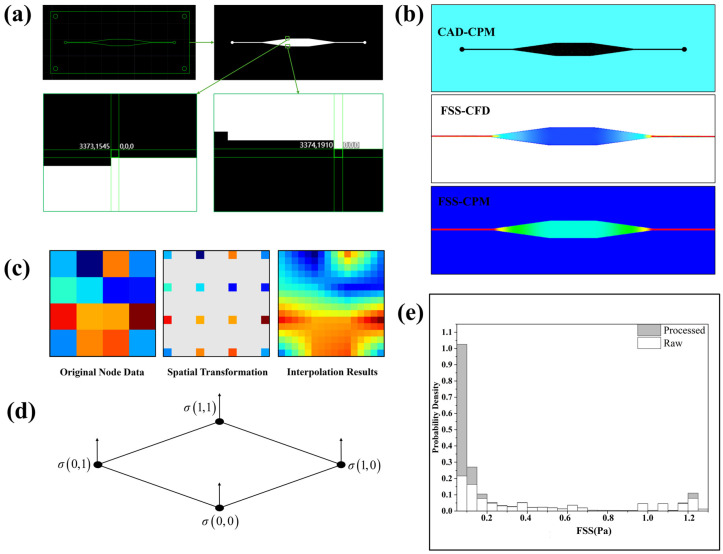
(**a**) Binary image of the chip. (**b**) Mapping result in CPM. (**c**) Pixels of different colors contain data from different nodes. The initial node is mapped to a high-resolution grid coordinate system containing numerous gaps to be filled. Using the values of the four surrounding known points, the value at the gap is calculated using a bilinear formula based on distance-weighted interpolation. (**d**) Bilinear interpolation surface. (**e**) Global distribution characteristics of FSS before and after interpolation.

To transform continuous biophysical processes into a computational model, the spatiotemporal domain of the simulation region was discretized by extracting pixel coordinates from binarized mask images of the chip (Figure 3a). Periodic boundaries were applied in the flow direction to ensure flow field continuity, whereas the physical channel walls were treated as frozen boundaries (Figure 3b). To address the resolution mismatch between the CFD mesh and the CPM lattice, a bilinear interpolation algorithm was employed to resample the flow field (Figure 3c,d) [27]. As shown in Figure 3e, the global FSS distribution became significantly smoother following interpolation, thereby providing a reliable data basis for accurate lattice-based dynamic evolution simulations.

#### 2.4.2. Dynamic Evolution Model of Osteoblasts Under FSS

In the computational model, FSS was not applied continuously. To accurately replicate intermittent physiological loading and align with in vitro experimental conditions, an intermittent loading paradigm was explicitly programmed into the CPM. By mapping the temporal scale such that 1440 Monte Carlo steps (MCS) represent a 24 h physiological day, the dynamic simulation applied the FSS field for 60 MCS (equivalent to 1 h), followed by a 1380 MCS (23 h) static resting phase for each simulated day.

The global simulation parameters and cellular morphology constraints are summarized in Table 1 and Table 2.

#### 2.4.3. Mechanobiological Rules for Cellular Evolution in CPM

Proliferation and Migration: The assumption of a constant cell division rate was discarded. Instead, the proliferation probability for each cell is jointly determined by its intrinsic basal probability, a spatial limitation factor (*ψ_s_*), and a shear-stress regulatory factor (*ψ_τ_*):

(6) $$\psi_{s}=\max\left(0,\frac{K-N}{K}\right)$$

(7) $$\psi_{\tau }(\tau )=\max(0,C_{0}+A\cdot \tanh[\alpha (\tau -\tau_{l})]-B\cdot \tanh[\beta (\tau -\tau_{h})])$$

where *N* and *K* denote the current local cell density and the maximum carrying capacity, respectively; *τ* represents the local FSS, with *τ_l_* and *τ_h_* defining its lower and upper thresholds; and *C*_0_, *A*, *B*, *α*, and *β* are empirical coefficients shaping the response curve.

When the population density approaches *K*, *ψ_s_* imposes a physical constraint reflecting contact inhibition. Concurrently, *ψ_τ_* employs hyperbolic tangent functions to accurately capture the nonlinear, biphasic “stimulation-damage” response to FSS. Furthermore, an adaptive migration-driving force field (*F_f_*) was constructed to propel the centroid of undifferentiated cells:

(8) $$F_{f}(\tau ,t)=\left\{\begin{array}{ll} \left[F_{M}\frac{\min(\tau ,\tau_{M})}{\tau_{M}}\right]\cdot \left(1-\frac{t}{t_{a}}\right)\cdot {\vec{u}}_{f} & ,t\leq t_{a}\\ 0 & ,t>t_{a} \end{array}\right.$$

where *F_M_* represents the maximal driving force magnitude, *τ_M_* is the shear-stress saturation threshold, *t_a_* denotes the cellular mechanosensory adaptation time, and $\vec{u_{f}}$ is the unit vector of the local flow direction.

Differentiation: Osteogenic differentiation is an irreversible process. To simulate the transition of cells that have completed their growth phase and entered differentiation under mechanical regulation, a nonlinear response function based on the Michaelis-Menten equation was adopted:

(9) $$\psi_{d}(\tau )=1.0+\alpha_{d}\frac{\tau -\tau_{c}}{K_{s}+(\tau -\tau_{c})}$$

where *α_d_* represents the maximum differentiation enhancement coefficient, *τ_c_* is the FSS activation threshold, and *K_s_* denotes the Michaelis constant corresponding to the half-maximal response. Furthermore, the initiation of differentiation requires the macroscopic spatial confluence of the system to reach a predefined threshold, thereby simulating contact inhibition.

Mineralization: Following differentiation, the osteoblasts enter the matrix secretion and mineralization stages. A two-stage decoupled mechanism was constructed. Initially, a local shear-stress regulatory coefficient, *ψ_m_*(*τ*), was introduced:

(10) $$\psi_{m}(\tau )=\left\{\begin{array}{ll} 1, & \mathrm{if}\;\tau <\tau_{c}\\ 1+\alpha_{m}\frac{\tau -\tau_{c}}{K_{s}+(\tau -\tau_{c})}, & \mathrm{if}\;\tau \geq \tau_{c} \end{array}\right.$$

where *α_m_* denotes the maximum enhancement amplitude for matrix secretion. Subsequently, cells with an osteoblastic phenotype secrete a collagen matrix into the surrounding lattice with a specific probability (*P_m_*):

(11) $$P_{m}(x,t)=P_{0}\cdot \psi_{m}(\tau )\cdot \Theta (t-t_{i}(\tau ))$$

where *P*_0_ is the basal secretion probability, Θ denotes the Heaviside step function, and *t_i_*(*τ*) represents the shear-dependent onset time of matrix secretion. Finally, terminal mineralization occurs exclusively when the local collagen concentration reaches a specific threshold and a nucleation region exists in the neighborhood. This process is governed by a strict logic gate:

(12) $$M=\Theta (C_{c}-C^{*})\cdot \Theta (t-t_{m})\cdot \Omega_{n}$$

where *C_c_* and C^*^ denote the local and threshold concentrations of the collagen matrix, respectively; *t_m_* is the maturation time required for mineralization; and Ω*_n_* represents the nucleation factor determined by the presence of existing mineralization cores in the local neighborhood. The mineralized regions exhibit significantly increased stiffness, thereby restricting further migration and deformation of surrounding cells.

## 3. Results and Discussion

### 3.1. Osteogenic Staining

The foundation of this mechanobiological response lies in the precise characterization of the mechanical microenvironment. Our CFD analysis demonstrated a broad, uniform FSS plateau (0.5–1.2 Pa) inside the microfluidic chip (Figure 2c), which falls well within the optimal range known to promote osteoprogenitor cell activity [25,26,32].

**Figure 4:**
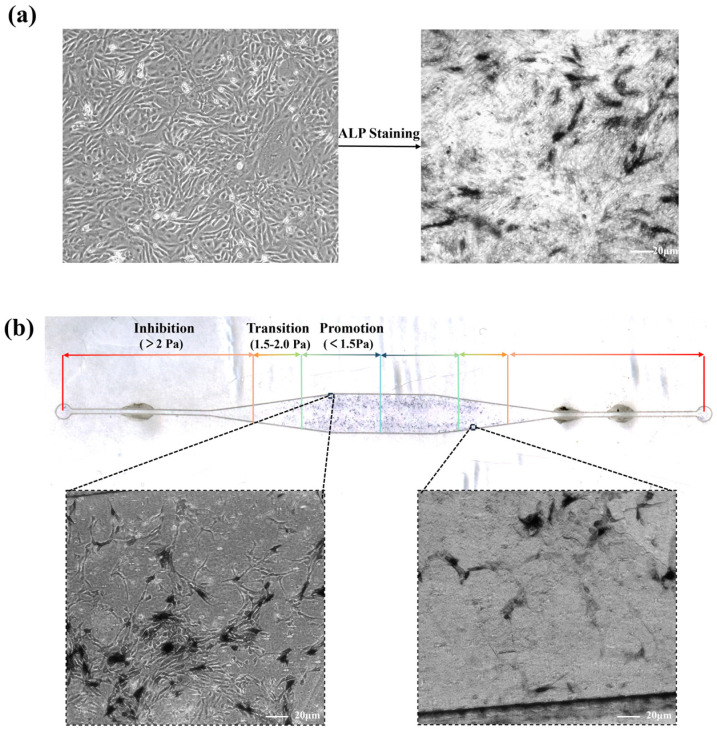
Staining outcomes of static culture and FSS groups: (**a**) ALP staining for osteogenic differentiation in the static group. (**b**) ALP staining for osteogenic differentiation within the microfluidic chip, the purple regions represent ALP-positive areas, indicating active osteogenic differentiation, and local microscopic images under different shear stresses were also obtained, where dark areas correspond to osteoblasts. The transition of the arrow colors from red to blue indicates a spatial decrease in fluid shear stress from high to low across the different regions of the chip.

Driven by this mechanical microenvironment, the conventional static culture exhibited a uniform, baseline expression of ALP, as shown in Figure 4a. In contrast, dynamic perfusion within the microfluidic chip induced a pronounced, spatially heterogeneous ALP distribution strongly correlated with the local FSS gradient (Figure 4b). Extensive purple ALP-positive regions and a high density of mature osteoblasts were observed in the central chamber, where the shear stress remained within the optimal range (<1.5 Pa).

This spatial correlation quantitatively aligns with the biphasic “stimulation-damage” theory of cellular mechanotransduction reported in previous independent studies, which demonstrated that moderate FSS promotes MC3T3-E1 proliferation and osteogenic marker expression, whereas stresses exceeding physiological limits trigger cell detachment and cell cycle arrest [5,6,29,33,34]. Consistent with these findings, our results demonstrated that both ALP staining intensity and the number of adherent cells were markedly reduced in the transition zones (1.5–2.0 Pa) and the narrow inlet and outlet channels (>2.0 Pa), corroborating that excessive shear stress induces mechanical fatigue rather than differentiation.

Furthermore, the successful induction of this targeted osteogenesis demonstrates that this rigid PET-based microfluidic architecture offers a highly reliable alternative to polydimethylsiloxane (PDMS), which is commonly used in organ-on-a-chip systems [23,24,35,36]. While PET chip fabrication relies on layer-by-layer ablation, making it potentially less versatile than PDMS for constructing highly complex 3D organ-on-a-chip structures [37], it remains entirely adequate for the requirements of the current study.

### 3.2. Cellular Dynamic Evolution Simulation Under FSS

**Figure 5:**
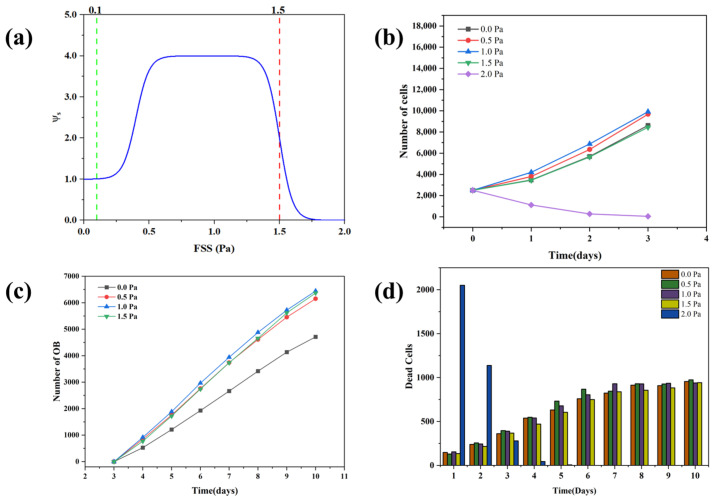
Growth simulation of MC3T3-E1 cells under 5 FSS levels: (**a**) The “stimulation–damage” curve regulated by FSS. (**b**) Overall cell apoptosis within 10 days. (**c**) Number of osteogenic differentiated cells during differentiation from day 3 to day 10. Cell differentiation under 2.0 Pa FSS was not analyzed, as most cells failed to survive to this stage. (**d**) Cell phase diagrams on day 10 under five shear stress intensities.

To accurately govern the mechanobiological response of the cells, the CPM incorporates a mathematically defined, nonlinear biphasic “stimulation-damage” function (Figure 5a). This regulatory curve dictates that moderate FSS yields a maximal biological gain plateau, whereas shear stress exceeding a critical threshold triggers a steep suppressive penalty. Driven by this underlying mechanism, the population evolution of MC3T3-E1 cells under five FSS levels (0.0–2.0 Pa) was simulated over a 10-day period. During the initial proliferation stage (days 0–3), FSS between 0.5 and 1.5 Pa promoted cellular growth compared to the ideal static control (0.0 Pa), peaking at an optimal stimulation of 1.0 Pa (Figure 5b). This aligns with experimental observations that FSS at approximately 0.8–1.2 Pa maximally upregulates osteogenic marker expression and proliferation in MC3T3-E1 cells [5,6,26]. Conversely, an excessive shear stress of 2.0 Pa induced severe growth inhibition and massive early apoptosis (Figure 5d), computationally corroborating prior reports that supra-physiological FSS triggers cellular damage, membrane compromise, and cell cycle arrest in osteoblasts [29,33,34].

Overall, the biphasic regulation of cellular behavior became highly apparent, echoing the dose-dependent “stimulation-inhibition” pattern observed in in vitro osteoblast cultures [34]. Our simulations successfully demonstrated that an appropriate FSS range (0.5–1.5 Pa) significantly enhanced proliferation [25,36], whereas excessive shear stress induced injury and cell cycle arrest [33,34].

Following the growth phase, the simulation transitioned to induced differentiation (days 3–10), excluding the non-viable 2.0 Pa group. The generation of mature osteoblasts exhibited a strong dependence on shear intensity; stimulation at 1.0 Pa yielded the maximum number of differentiated cells, while 0.5 Pa and 1.5 Pa provided moderate enhancements over the static baseline (Figure 5c). This shear-intensity-dependent differentiation profile agrees with experimental studies showing that moderate FSS maximally induces osteogenic marker expression and mineralization in osteoblasts [5,26,38], and that optimizing flow rates is critical for maximizing mineralized matrix production [39].

### 3.3. Spatio-Temporal Evolution of Cellular Morphology and Proliferation

**Figure 6:**
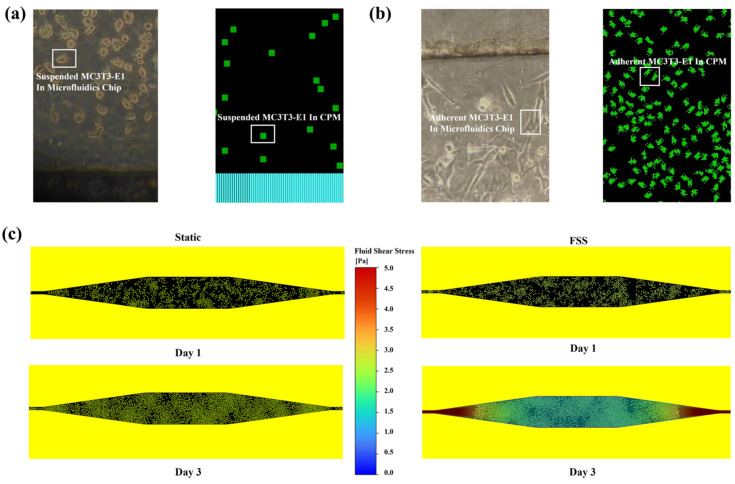
FSS-driven cell proliferation fate decision model and early spatio-temporal evolution characteristics in microfluidic chip: (**a**) Captured images of cells in the chip at suspended state immediately after seeding; the initial suspended cell morphology in the corresponding CPM on the right is represented by quadrilaterals. (**b**) The left image shows cells in adherent state inside the chip, presenting a fusiform shape with actin-based pseudopodia. The MC3T3-E1 cells imposed with major axis constraints in the right CPM possess analogous pseudopodial structures. (**c**) Spatio-temporal evolution snapshots of the cell population during the early proliferation stage.

To establish a rigorous correspondence between the computational framework and actual physiological time, the simulation mapped 1440 Monte Carlo steps (MCS) to a 24 h cycle, of which 60 MCS corresponded to the 1 h daily FSS exposure and the remaining 1380 MCS represented the static incubation period, consistent with the intermittent perfusion protocol (1 h/day) used in the in vitro experiments [5,26,32]. The spatiotemporal evolution during the early proliferation phase (days 1–3) revealed that population dynamics were highly dependent on the macroscopic mechanical field. Specifically, the static control group (0.0 Pa) was computationally configured to exclude mechanical disturbances associated with medium exchange. This established an idealized baseline that consequently exhibited uniform spatial growth (Figure 6c).

In contrast, the spatial distribution under dynamic FSS displayed an FSS-dependent state transition, consistent with experimental observations from multi-shear microfluidic platforms demonstrating that osteoblast responses are spatially heterogeneous under varying FSS magnitudes [24,36]. In the central chamber, where the fluid field formed a uniform plateau within the optimal stimulation regime, osteoprogenitor cells maintained a robust proliferative state [5,6]. These local aggregates continued to divide and expand until reaching a volumetric limit, which was regulated by the spatial dimensions of the microchamber and the algorithm’s contact inhibition constraints [21,40]. Conversely, when the local FSS exceeded the critical tolerance threshold (>2.0 Pa), a distinct fate transition occurred. The osteoprogenitor cells located in the narrow inlet and outlet channels accumulated mechanical damage, rapidly transitioning from a proliferative state into one characterized by apoptosis and substrate detachment. This heterogeneous spatial distribution indicates that maintaining FSS within an appropriate physiological window is important for the sustainable growth and functional population expansion of osteoblasts in microfluidic systems.

### 3.4. Osteogenic Differentiation Simulation

**Figure 7:**
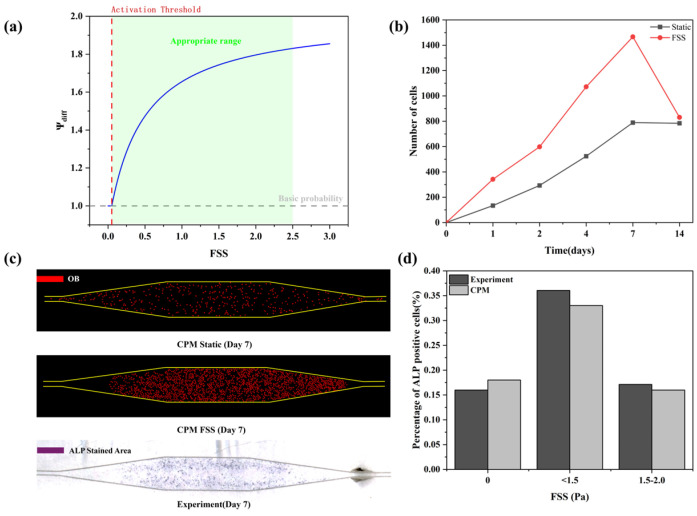
Differentiation period: (**a**) Differentiation gain factor. (**b**) Number of progenitor cells within 14 days after induced differentiation. (**c**) Comparison of CPM simulation images and experimental staining results on day 7 of induced differentiation under static state (without FSS) and FSS loading. Undifferentiated precursor cells were masked to better observe osteogenic differentiation. (**d**) Proportion of ALP-positive cells in regions with varying fluid shear stress levels.

Following the initial growth phase, the transition of osteoprogenitor cells into mature osteoblasts was simulated over a 14-day period, governed by the differentiation gain factor, ψ*_diff_* (Figure 7a). To model this irreversible transition, a Michaelis-Menten-based nonlinear response function under local mechanical stimulation was employed. Within the CPM lattice, the local fluid shear stress *τ* acting on individual cells modulates their basal differentiation probability. Once this mechanical stimulus exceeds a predefined activation threshold, the cellular differentiation rate follows a saturation curve, reflecting the limited receptor-mediated mechanotransduction capacity inherent to biological systems. This mathematical formulation aligns with the multiphase modeling framework proposed by Pearson [31], who applied Michaelis-Menten-type kinetics to simulate nonlinear cellular fate decisions under dynamic fluid flow.

The temporal dynamics of the progenitor population (Figure 7b) indicated that while the static group exhibited a gradual trajectory, the FSS-loaded group peaked at day 7. Driven by local flow gradients, regions experiencing higher shear stress exhibited accelerated differentiation. This trend is consistent with the well-documented temporal progression of MC3T3-E1 differentiation, in which progenitor cells are progressively depleted as they commit to the osteoblast lineage [41].

The spatial heterogeneity of this transition is illustrated in the day 7 CPM phase diagrams (Figure 7c). With undifferentiated precursors masked, the static simulation displayed a sparse, random distribution of mature osteoblasts (red). In comparison, the FSS-driven simulation demonstrated an accumulation of osteoblasts concentrated within the optimal shear regions of the central chamber. This simulated spatial pattern is comparable to our experimental results; the macroscopic distribution of osteoblasts predicted by the CPM closely corresponds to the ALP-positive regions observed in the microfluidic chip.

To evaluate the predictive accuracy of the computational model, a quantitative comparison of ALP-positive cell proportions across varying FSS zones was conducted (Figure 7d). The computational predictions showed strong agreement with the in vitro experimental data. Specifically, both the simulation and the experiment indicated that the proportion of differentiated cells peaked within the optimal stimulation regime (<1.5 Pa), while differentiation efficiency was markedly reduced in the transition zones (1.5–2.0 Pa) and the static control group (0 Pa). This quantitative concordance suggests that the constructed spatial Michaelis-Menten rules effectively capture the mechanosensitivity and fate decision mechanisms of osteoprogenitor populations.

### 3.5. Terminal Mineralization Simulation

**Figure 8:**
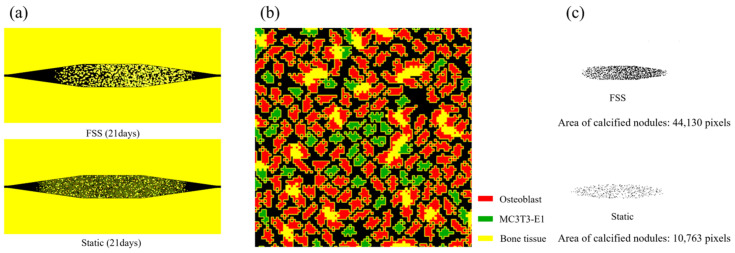
Characteristics of terminal mineralization distribution and morphological evolution during bone formation: (**a**) Comparison of global spatial distribution of calcified nodules in microfluidic culture regions between FSS group and static group. (**b**) Local lattice state snapshots in the core region of CPM simulation. (**c**) Macroscopic contours of actual mineralized nodules after binarization extraction.

To evaluate the spatial distribution of calcified nodules, a CPM analysis was conducted at day 21. By extrapolating the mechanobiological rules calibrated from the 7-day early differentiation experiments, the model enables the predictive simulation of long-term terminal mineralization, thereby circumventing the technical bottlenecks typically associated with prolonged 21-day microfluidic perfusion cultures. As shown in Figure 8a, macroscopic bone-like tissue structures were formed in both dynamic and static simulations. However, the FSS-driven simulation exhibited enhanced matrix deposition. Following image binarization (Figure 8c), the total calcified nodule area in the FSS group reached 44,130 pixels, notably exceeding the 10,763 pixels observed in the idealized static control group. This approximately fourfold enhancement is consistent with in vitro perfusion bioreactor studies: Bancroft et al. [42] reported that all flow conditions yielded significantly higher mineralized matrix production than static controls, with calcium content increasing in a dose-dependent manner with flow rate, while Sikavitsas et al. [43] demonstrated that increasing fluid shear forces—while holding chemotransport constant—directly increased mineral deposition. Datta et al. [44] further showed that FSS and bone-like extracellular matrix act synergistically, producing an up to 75-fold increase in calcium content. The strong agreement between our simulation results and these experimental findings suggests that the mechanobiological rules captured by the CPM adequately represent the osteoinductive effect of shear stress on terminal mineralization.

Unlike previous cellular automata models that were limited to static cultures without mechanical cues [13,45,46], our model introduces FSS as an active inducing factor for terminal bone matrix formation [4,38,44]. Under this framework, regions with dynamic, uniformly distributed shear stress achieved quantitatively higher and more stable matrix deposition [47] with a more uniform spatial distribution [39,43]. This is consistent with perfusion bioreactor studies showing that FSS enhances both the amount and the spatial distribution of the mineralized matrix, whereas static culture produces heterogeneous, surface-localized deposition [42,43]. Local lattice snapshots (Figure 8b) further reveal that mature bone tissue (yellow) accumulated around differentiated osteoblasts (red), while undifferentiated MC3T3-E1 cells (green) were confined to peripheral, low-shear regions. This spatial segregation mirrors the stage-dependent compartmentalization of MC3T3-E1 cultures, in which proliferating cells remain ALP-negative and non-mineralizing until growth arrest initiates matrix deposition [41]. Furthermore, it aligns with bioreactor observations demonstrating that dynamic perfusion drives cell differentiation into the scaffold interior, while static conditions leave undifferentiated cells at the periphery [48].

## 4. Conclusions

This study establishes a multiscale computational framework integrating fluid mechanics with a cellular Potts model to simulate the mechanobiological evolution of osteoprogenitor cells. While existing approaches have advanced mechanobiology, they generally lack the integration of computational fluid dynamics with the CPM. Our framework addresses this methodological gap, establishing a direct mechanistic link between complex shear stress fields and cellular evolution. The integrated platform successfully translates the mesoscale perception of physical forces into macroscopic biological responses, accurately reproducing the nonlinear biophysical dynamics where controlled FSS guides cell proliferation and differentiation, and predicting spatial matrix mineralization. The strong agreement between the in silico spatial patterns and in vitro microfluidic staining validates the model’s robust capability to capture the mechanically driven self-organization mechanism inherent to early bone remodeling.

While the long-term mineralization predicted by the model is well-corroborated by established literature, direct in vitro validation (such as Alizarin Red staining) was not performed in this study. Specifically, although the thin-film PET chip employed facilitates rapid fabrication and reliable early differentiation assessment, incorporating such endpoint assays would further enhance the biological fidelity of the model by providing direct physicochemical confirmation of matrix mineralization. Maintaining stable long-term perfusion cultures poses inherent technical challenges compared to mature PDMS-based platforms. Consequently, optimizing the microfluidic device for prolonged dynamic culture and completing the terminal experimental validation should be performed in future investigations. Furthermore, a comprehensive understanding of bone homeostasis requires extending this description beyond isolated monocultures toward a framework that accounts for 3D architectural constraints and multi-cellular crosstalk. We emphasize that the representation of complex in vivo bone microenvironments within this in vitro framework remains at a preliminary stage of development. Direct simulation evidence incorporating full 3D osteoclast-osteoblast coupling is still evolving, and our current 2D mesoscale approach should therefore be seen as a robust foundation that complements future comprehensive tissue-level digital twin models.

## Figures and Tables

**Table 1:** Global Simulation Parameters.

Parameter	Value/Setting	Unit	Description
*S*	31,680	/	Total number of Monte Carlo steps [28]
*T*	10.0	/	Activity of membrane edge fluctuation [20]
*τ*	/	Pa	Dynamic mechanical stimulation
*τ_l_*	0.5	Pa	Low FSS threshold [29]
*τ_h_*	2.0	Pa	High FSS threshold [29]
*C*_0_	0.2	/	Basal proliferation regulatory constant
*A*	0.8	/	Proliferation enhancement coefficient
*B*	1.2	/	Proliferation suppression coefficient
*α*	0.5	/	Decline rate of cell activity slope
*β*	0.8		Response slope for high shear damage
*τ_m_*	2.5	Pa	Shear stress saturation threshold [29]
*F_M_*	150.0	/	Maximal driving force magnitude
*t* *_a_*	30	MCS	Mechanosensory adaptation time [30]
*δ*	0–1.0	/	Cumulative cell damage value
*α_d_*	6.0	/	Differentiation enhancement coefficient
*α_m_*	5.0	/	Matrix secretion enhancement coefficient
*K_s_*	0.5	/	Michaelis constant [31]
*R*_0_	0.32	μm^3^/MCS	Basal matrix secretion rate [5]

The dimensionless mathematical coefficients ($C_{0},\;A,\;B,\;\alpha ,\;\beta ,{\;\alpha }_{d},{\;\alpha }_{m}$) and driving force magnitude ($F_{M}$) are empirical tuning parameters within the CPM framework. They were iteratively calibrated in the present study to ensure that the computational nonlinear response curves accurately replicate the qualitative biphasic cell fate trends observed in typical in vitro microfluidic experiments.

**Table 2:** Cell Morphological Constraints.

Cell	*V_T_*	*λ* *_v_*	*S_T_*	*λ* *s*	*L_T_*	*λ* *_l_*
MC3T3-E1	16	15	28	3	8.5	5
Osteoblast	20	15	30	3	10	5

*V_T_***:** target volume; *λ_v_***:** volume elasticity coefficient; *S_T_*: target surface area; *λ_s_*: surface area elasticity coefficient; *L_T_*: target length; *λ_l_*: length elasticity coefficient. The values were determined based on the actual cell size.

## Data Availability

The datasets in this article are available from the corresponding author upon reasonable request.
